# Transgender people and *travestis* experiencing homelessness in Salvador: a descriptive study on sociodemographic profile and access to social assistance and health services, Brazil, 2021 and 2022

**DOI:** 10.1590/S2237-96222024v33e2024515.especial.en

**Published:** 2025-01-13

**Authors:** Wiler de Paula Dias, Thayane Silva Nunes, Igor Myron Ribeiro Nascimento, Erik Asley Ferreira Abade, Lua Sá Dultra, Milena Lemos Marinho, Guilherme de Sousa Ribeiro, Joilda Silva Nery

**Affiliations:** 1 Universidade Federal da Bahia, Instituto de Saúde Coletiva, Salvador, BA, Brasil; 2 Universidade Federal da Bahia, Faculdade de Medicina, Salvador, BA, Brasil; 3 Fundação Oswaldo Cruz, Instituto Gonçalo Moniz, Salvador, BA, Brasil

**Keywords:** Personas en Situación de Calle, Personas Transgénero, Travestis, Vulnerabilidad Social, Encuesta Epidemiológica, Homeless Population, Transgender People, Travestis, Social Vulnerability, Epidemiological Survey

## Abstract

**Objective:**

To describe the sociodemographic profile and access to social assistance and health services among trans people and *travestis* experiencing homelessness in Salvador, the capital city of Bahia state.

**Methods:**

This was a cross-sectional study involving 24 (4.5%) participants who identified as trans people or *travestis* out of a total of 529 people, aged 18 or older, living in public spaces or institutional shelters included in the survey. Data were collected between 2021 and 2022, using structured questionnaires.

**Results:**

The sample was predominantly comprised of trans women and *travestis* (n=18), young individuals (n=13), of Black race/skin color (n=22), single individuals (n=18) and those engaged in informal occupations (n=23), many of whom reported experiencing violence (n=17). Frequent barriers to accessing healthcare and social assistance services were found (n=15), including lack of documentation, delay in service provision and social/racial discrimination.

**Conclusion:**

The findings highlight the heightened vulnerability of transgender people and *travestis* experiencing homelessness, who are subjected to multiple forms of discrimination and social exclusion.

## Study contributions

**Main results:**

The sample profile was comprised of young, Black, single transgender women engaged in informal employment. The primary barriers to accessing health and social assistance services were the lack of identification documents and experiences of social and racial discrimination.

**Implications for services:**

The study addressed the disconnection and barriers faced by transgender people and *travestis* in accessing health and social assistance services. It emphasized the need to strengthen strategies aimed at expanding care networks and ensuring appropriate support for this population.

**Perspectives:**

Further studies are recommended to investigate the potential increased vulnerability of transgender people and *travestis* to homelessness and their health needs. The urgent implementation of public policies tailored to meet the demands of this population is critical.

## RESUMEN

**Objetivo:**

Describir el perfil sociodemográfico y el acceso a los servicios socioasistenciales y de salud entre personas trans y travestis en situación de calle en Salvador.

**Métodos:**

Se trata de un estudio transversal con 24 participantes (4,5%) que se autoidentificaron como personas trans o travestis, del total de 529 personas con edad igual o superior a 18 años, presentes en espacios públicos o unidades de acogida institucional incluidas en el estudio. Los datos fueron recolectados entre 2021 y 2022 mediante cuestionarios estructurados.

**Resultados:**

La muestra estuvo compuesta mayoritariamente por mujeres trans y travestis (n=18), jóvenes (n=13), negras (n=22), solteras (n=18) y con ocupaciones informales (n=23), quienes reportaron haber sufrido violencia (n=17). Se encontraron dificultades frecuentes para acceder a los servicios de salud y socioasistenciales (n=15), principalmente debido a la falta de documentación, largas demoras en la atención y discriminación social/racial.

**Conclusión:**

Se observa una marcada vulnerabilización de las personas trans y travestis en situación de calle, quienes están sometidas a múltiples formas de discriminación y exclusión social.

## INTRODUCTION

The homeless population is defined as a heterogeneous group that shares the intersection of structural and personal factors, such as economic exclusion, racism, severed family ties, and the absence of regular conventional housing, relying on the streets as their primary space for social reproduction^.1,^ 2 The existence and growth of the homeless population are not recent phenomena. They reflect historical inequalities and macroeconomic issues that persist as consequence of systemic factors in the capitalist system^.3-^ 5

In Brazil, in 2023, it was estimated that over 236,000 people lacked permanent residence, living on the streets or in shelters for the homeless population. The population profile was predominantly comprised of adults aged 30 to 49 years (55%), male (87%), Black individuals (68%) and those with previous experience of employment contract (68%)^.^ 6

The rupture with the cis-heteronormative standard contributes significantly to social disengagement and increases the likelihood of homelessness among the LGBTQIAPN+ population, especially for trans people and *travestis* ^.7-^ 9 Family conflicts related to nonconformity with the binary sex-gender system, are cited as the main reason for estrangement. This reinforces discrimination based on sexual orientation and gender identity as a social determinant of health^.10-^ 12

Non-cisgender people face vulnerabilities within both the family environment and on the streets, leading to lifelong exclusion and denial. These people are subjected to contexts of dehumanization and abjection, as well as a greater exposure for physical and sexual violence^.8,9,10,^ 13 In 2023, in Brazil, 136 cases of murders of *travestis* and trans women were reported, with a focus on Black women, aged 18 to 29 years, many of whom were sex workers, mainly in the Northeast and Southeast regions of the country^.^ 14 The lack of secure housing further exacerbated their vulnerability to violence^.13,^ 15

In the health sector, significant challenges persist that hinder access to services and compromise the quality of healthcare provided. Insufficient training of professionals regarding the health needs of the LGBTQIAPN+ population, enduring social prejudice against the homeless population, in addition to the stigma associated with the human immunodeficiency virus (HIV), are among the factors that may increase this population’s vulnerability to health conditions, diseases, and unfavorable health outcomes^.10,11,16,^ 17 Research on the health conditions and access barriers faced by transgender individuals and *travestis* remains scarce, particularly in highly vulnerable contexts such as homelessness, where efforts to promote the physical and mental well-being of this population are critical^.10,^ 17

This study aimed to contribute to the construction of knowledge on the subject. It sought to describe the sociodemographic profile and access to social assistance and health services among transgender people and *travestis* experiencing homelessness in Salvador, Bahia state, between 2021 and 2022.

## METHODS

### Study design

This work was derived from the epidemiological survey Health Conditions of the Homeless Population in Salvador – Bahia state, 2021, a cross-sectional study that included a sample of homeless people in Salvador, Bahia state.

### Setting

**Figure 1:**
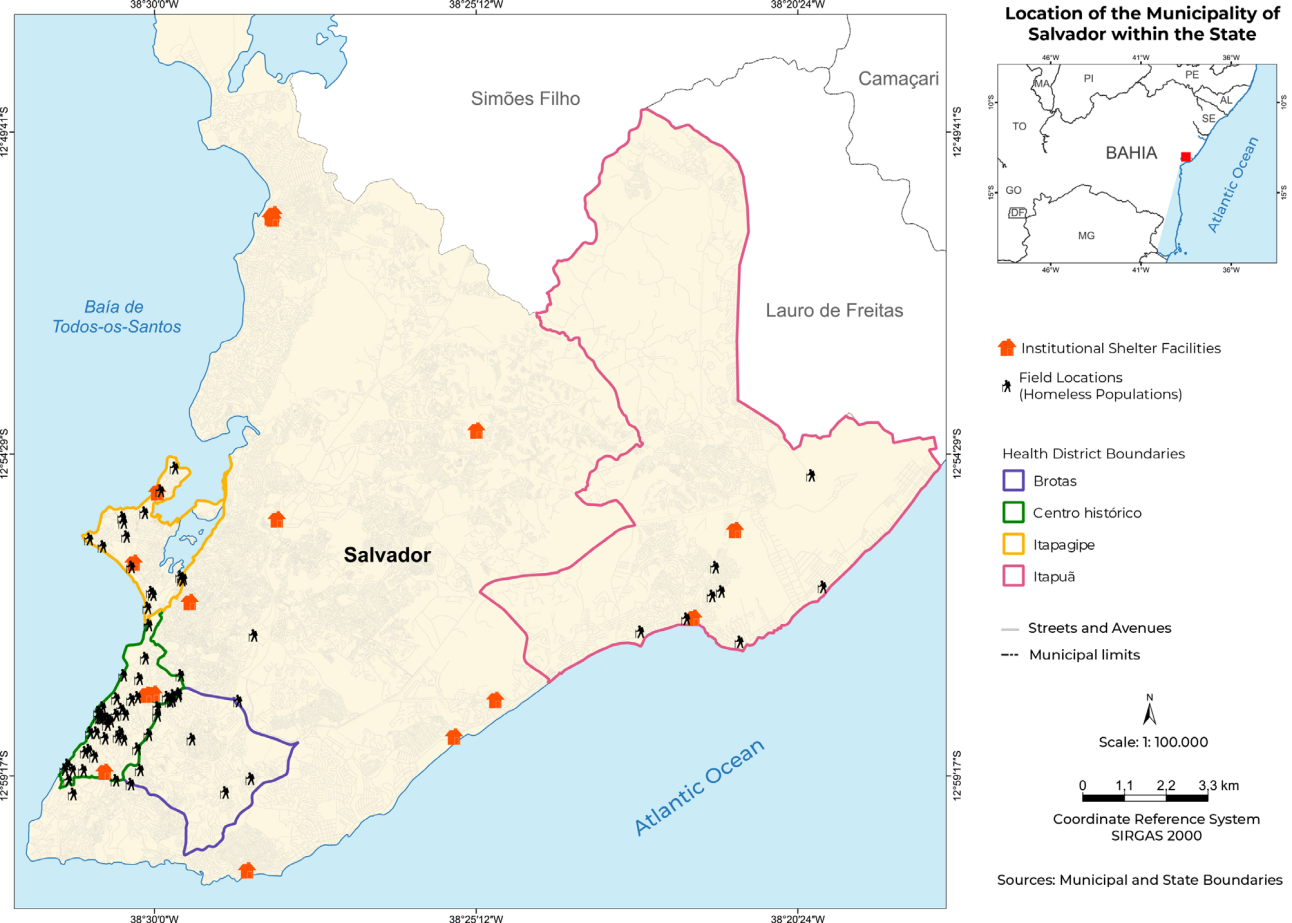
Coverage of health districts, points of concentration of the homeless population and institutional shelters, Salvador, Brazil, 2021-2022

Data collection for the survey took place between September 2021 and February 2022. Participant inclusion sites were defined after identifying institutional shelters associated with the Municipal Department of Social Promotion, Poverty Combat, Sports, and Leisure of Salvador (17 institutional shelters) and mapping public spaces (40 locations) where homeless populations concentrate and where the city’s five street clinic teams operate: Centro Histórico-Gamboa, Centro Histórico-Pelourinho, Itapuã, Itapagipe, and Brotas (Figure 1).

The institutional shelters in Salvador were categorized by types (female, male, mixed, couples, older adults and families). In order to ensure diversity among the homeless population interviewed, given their heterogeneous nature, at least one institutional shelter of each type was included in the study, selected through random sampling by subtype. Two to three institutional shelters were defined for each street clinic team, in a predetermined order.

For street-based sampling, public spaces with a concentration of homeless individuals were randomly selected for each street clinic team, with a priority order defined for conducting the survey. All identified points were visited across all street clinic areas. As the minimum number of participants was not reached during the first visit to each location, repeated visits were conducted following the prioritization protocol. Since this is a hard-to-reach population, all interested individuals present at the sites were offered participation, provided they met the predefined eligibility criteria.

### Participants

The target reference population for inclusion in the survey consisted of people identified as homeless. Their characteristics corresponded to those described in the National Policy for the Homeless Population: a heterogeneous population group living in extreme poverty, with severed or weakened family ties, lacking permanent housing, and “using public spaces and degraded areas as living and livelihood spaces, temporarily or permanently, as well as shelters for temporary or provisional housing”^.^ 1

The eligibility criteria for participating in the study included: self-identification as a homeless person, as per the aforementioned definition, absence of intellectual disability (permanent or transient), whether associated with psychoactive substance use or not, that would impair the ability to respond to the questionnaire, and absence of respiratory symptoms within the past 10 days to mitigate the risk of COVID-19 transmission to the research team. Participants with respiratory symptoms were referred for care following the municipal protocol in place at the time.

### Variables

For analyses, variables were grouped into three large blocks, as follows:

Sociodemographic and economic variables: gender (trans woman, *travesti*, trans man, non-binary person), age group (18-29 years, 30-39 years, 40-49 years, ≥50 years), race/skin color (Black, White, mixed-race, Asian, Indigenous), sexual orientation (heterosexual, gay , lesbian, bisexual, asexual, pansexual), education level (complete/incomplete higher education, complete/incomplete high school, complete/incomplete elementary school, no education), can read (no; yes, without difficulty; yes, with difficulty), can write (no; yes, without difficulty; yes, with difficulty), marital status (single, legally married, in a stable union, separated or divorced, widowed), place of birth (Salvador and the Metropolitan Region of Salvador, other locations), time as a homeless person (<1 year, 1-4 years, 5-9 years, ≥10 years), monthly income (<¼ minimum wage, ¼-½ minimum wage, ½-¾ minimum wage), disability status (no, yes) and health district (Centro Histórico, Itapuã, Itapagipe, Brotas).
Occupational variables: currently employed (no, yes), engaged in informal work (no, yes), number of informal jobs simultaneously (1, 2, ≥3), work on the books (yes, currently; yes, in the past; never), ever barred from working (no, yes) and experienced workplace violence (no, yes).
Access to social assistance and health services: history of institutional sheltering (no, yes), registered in the Unified Registry (no, yes), Bolsa Família beneficiary (no, yes), difficulty accessing healthcare centers (no, yes), usual place of care (polytomous variable), barriers to accessing health services (polytomous variable), social assistance services accessed in the past year (polytomous variable), follow-up in mental health services (polytomous variable) and health care received on the street (polytomous variable).

### Data sources and measurement

Data collection was performed by a team comprised of professionals from street clinics, workers from mental health, human rights services and social assistance staff, university students and volunteers. After participants’ consent, the questionnaires were administered using electronic devices (cell phones and tablets), through the Research Electronic Data Capture software (RedCap).

### Study size

The final sample for this study consisted of 24 participants (4.5%) who identified as trans people or *travestis*, out of a total of 529 individuals included in the survey on homeless people. Inclusion in the sample was based on the question “How do you identify in terms of gender? (What is your gender identity?)”. Valid responses included: trans woman, *travesti*, trans man, non-binary people and others.

The minimum sample size for the survey on homeless people was calculated at 560 participants, based on an estimated 50% prevalence for multiple health outcomes, a 95% confidence level and a sampling error of 4%, without design effect adjustments.

### Data analysis

A descriptive analysis of participant profiles was performed using absolute frequencies, by category. Database organization and cleaning, as well as statistical analyses, were conducted using R software, version 4.2.3 (x64). Illustrations were created by the authors, based on data from the Health Conditions of the Homeless Population in Salvador survey – Bahia state, 2021.

### Ethical aspects

The study was conducted in accordance with the recommendations of Resolution No. 466/2012 of the National Health Council, and was approved by the Research Ethics Committee of the Instituto Gonçalo Moniz /Fiocruz (CAAE: 42517021.0.0000.0040), and Opinion No. 5,138,407, approved on December 1, 2021. Participants were informed about the objectives, procedures, and risks. Upon agreeing to participate in the survey, they signed and received a copy of the free informed consent form.

## RESULTS

Among the 529 homeless people interviewed, 24 identified as transgender people and *travestis*, representing 4.5% of the total sample. The transgender and *travesti* population experiencing homelessness included predominantly transgender women (n=15), young adults aged 18 to 29 years (n=13), heterosexual individuals (n=12), self-identified Black and mixed-race persons (n=22), single individuals (n=18), those with an average monthly income of up to half the minimum wage (n=9), individuals from Salvador or the metropolitan region (n=20), and those without physical or mental disabilities (n=21). The majority had completed or incomplete elementary school (n=14) . Many had been homeless for less than a year (n=14) . The Centro Histórico health district concentrated the largest number of respondents (n=11) (Table 1).

**Table 1:** Description of transgender people and travestis experiencing homelessness according to socioeconomic and demographic characteristics, Salvador, Brazil, 2021-2022 (n=24)

**Variables**	**n**
Self-reported gender	
Trans woman	15
*Travesti*	3
trans man	4
Non-binary person	2
**Age range (years)**	
18-29	13
30-39	6
40-49	4
≥50	1
**Race/skin color**	
Black	14
White	2
Mixed-race	8
**Self-reported sexual orientation**	
Heterosexual	12
Gay	6
Lesbian	2
Bisexual	2
Asexual	1
Pansexual	1
**Education level**	
No formal education	-
Complete or incomplete elementary school	14
Complete or incomplete high school	8
Complete or incomplete higher education	2
**Reading ability**	
Cannot read	3
Can read with difficulties	5
Can read without difficulties	16
**Writing ability**	
Cannot write	1
Can write with difficulties	9
Can write without difficulties	14
**Marital status**	
Single	18
Legally married	-
Stable union	4
Separated or divorced	1
Widowed	1
**Place of birth**	
Salvador and metropolitan region of Salvador	20
Other location	4
**Time spent homeless (years)**	
<1	14
1-4	3
5-9	3
≥10	4
**Monthly income^a^(minimum wage)**	
<¼	2
¼- ½	7
½-¾	1
> ¾	4
**Have a disability ^b^**	
No	21
Yes	2
**Health district**	
Centro Histórico	11
Itapuã	5
Itapagipe	4
Brotas	4

a) Based on the minimum wage in 2021; b) Absolute response values vary due to missing data (blank responses) for the analyzed variable (n=23).

More than half (n=14) of the transgender and *travesti* population experiencing homelessness reported having stayed in institutional shelter facilities. Fifteen people indicated registration in the Unified Registry for Social Programs. Most participants were not Bolsa Família beneficiaries (n=15). Within the 12 months prior to the survey, the most frequently accessed services included the Specialized Reference Center for the Homeless Population (n=13), followed by the Social Assistance Reference Center (n=10), the Specialized Social Assistance Reference Center (n=2) and the Social Interaction Center (n=2).

Eighteen people reported being unemployed, yet high participation in informal activities (n=23) was observed. Regarding frequency, most participants engaged in only one informal activity at a time (n=18). The majority had never held formal employment (n=20). A significant proportion (n=17) of trans people and *travestis* reported experiencing some type of aggression, threat or violence while performing their work activities (Table 2).

**Table 2:** Description of transgender people and travestis experiencing homelessness by occupational and social assistance service variables, Salvador, Brazil, 2021-2022 (n=24)

Variables	n
**Currently working**	
No	18
Yes	6
**Engaged in informal work**	
No	1
Yes	23
**Number of simultaneous informal jobs**	
1	18
2	4
≥3	1
**Have worked with formal employment**	
No (never)	20
Yes (in the past)	4
Yes (currently)	-
**Prevented from working**	
No	15
Yes	9
**Experienced violence while working**	
No	7
Yes	17
**Types of informal work performed** ^b^	
Waste picker	5
Sex worker	10
Car guard/cleaner	3
Sales person	8
General services	1
Manicurist and pedicurist	2
**Social assistance services accessed in the past year** ^b^	
CRAS	10
CREAS	2
Centro POP	13
Social and community bonding center	2
None	2
**Institutional sheltering history**	
No	10
Yes	14
**Registration in the Unified Registry**	
No	9
Yes	15
**Bolsa Família beneficiary**	
No	15
Yes	9

a) Absolute response values vary due to missing data (blank responses) for the analyzed variable (n=23); b) N total reflects the possibility of multiple response options; c) Social Assistance Reference Center (*Centro de Referência da Assistência Social-* CRAS); Specialized Social Assistance Reference Center (*centro de referência especializado da assistência social* – CREAS); Specialized Reference Center for the Homeless Population (*centro de referência especializado para população em situação de rua* - Centro POP).

Findings related to access to health services among trans people and *travestis* experiencing homelessness indicate that the emergency care units were the most common facilities sought for medical services (n=19), followed by street clinics (n=9) and primary healthcare centers and family health units (n=8), while hospitals were the least frequently accessed (n=4). There were no reports of seeking care in polyclinics or specialized units. The majority indicated encountering barriers to accessing healthcare facilities (n=12). The reported reasons for these barriers included the absence of identification documents (n=3), lack of a national health card (n=5), and long wait times for care (n=6). Social (n=4) and racial (n=1) discrimination were also identified as obstacles (Table 3).

**Table 3:** Description of transgender people and travestis experiencing homelessness according to access to healthcare services, Salvador, Brazil, 2021-2022 (n=24)

Variables	n
**Faced difficulty accessing healthcare centers** ^a^	
No	11
Yes	12
**Usual healthcare access points** ^b^	
PHC/FHU	8
CnaR	9
Emergency care unit	19
**Polyclinics or specialty centers**	
Hospitals	4
Does not usually access healthcare services	-
Others^c^	1
**Barriers to accessing healthcare services** ^b^	
Lack of identification document	3
Lack of Brazilian National Health System card	5
No prior appointment scheduling	1
Racial discrimination	1
Social discrimination	4
Long waiting times	6
**Mental health service history** ^b^	
Never	7
CAPSad or CAPSad III	7
CAPSia /CAPS II/CAPS III	1
Therapeutic community or recovery center	5
Hospitalization or Cata	1
Outpatient clinic or Cetad	2
Others^d^	1
**Healthcare services received on the street** ^b^	
Medical or nursing consultations	12
Vaccination	7
Medication administration	3
Wound care	4
Rapid testing for STIs	7
Sputum collection for tuberculosis investigation	2
Care by health professionals (excluding doctors or nurses)	9

a) Dichotomous variable. Absolute response values varied due to missing data (blank responses) for the analyzed variable (n=23). b) The total N reflects the possibility of multiple response options. c)Medical school. d) Harm reduction specialists, psychologists, occupational therapists, social workers, and physical educators. e) PHC/FHU – Primary Healthcare Center or Family Health Unit; Street Clinic (*consultório na rua* – CnaR); Psychosocial Care Center for Alcohol and Drugs (*centro de atenção psicossocial álcool e drogas* – CAPSad); Psychosocial Care Center for Children and Adolescents (*centro de atenção psicossocial infância e adolescência* – CAPSia); Center for Alcohol Treatment and Recovery (*centro de acolhimento e tratamento de alcoolistas* – Cata); Center for Studies and Therapy of Drug Abuse; STIs – Sexually Transmitted Infections (*centro de estudos e terapia do abuso de drogas; e ISTs – infecções sexualmente transmissíveis* – Cetad).

Many participants had never accessed mental health services (n=7). An equal number (n=7) reported receiving care from psychosocial support centers specializing in alcohol and other drug use. Therapeutic communities or recovery centers related to alcohol and other drug abuse (n=5) were also used.

Street-based healthcare services most commonly involved medical or nursing consultations (n=12). Care provided by other categories of health professionals also stood out. Vaccination (n=7), rapid testing for sexually transmitted infections (n=7) and administration of medication (n=3) were among the most frequent procedures provided.

## DISCUSSION

The findings of this research highlight a greater vulnerability of trans women and *travestis* to violence and unfavorable social outcomes, even when compared to other subgroups within the LGBTQIAPN+ population^.7,8,9,11,^ 14

It was estimated that 1.9% of the adult population of Brazil identifies as trans people or *travestis* ^.^ 18 The 4.5% proportion observed in this study underscores the impact of social exclusion experienced by transgender people and *travestis* living in street situations, a consequence of the social marginalization resulting from their divergence from cis-heteronormative expectations^.7-9,11,12,^ 17

From gestation to the moment they express their identity and challenge the sex-gender system and its associated behavioral expectations, transgender people and *travestis* are rendered invisible, perceived as “the other,” “the stranger,” or “the abject,” existing solely to be erased^.^ 13 “It is a process of granting life, through discourse, only to extinguish it immediately”^.^ 19

Most trans people and *travestis* identified as Black, predominantly Black people. This data reflected the historical composition of the homeless population, referring to former enslaved individuals. Following the abolition of slavery, these people were left without legal or reparative support and turned to public spaces, particularly urban areas, for residence^.4,^ 5 Although being part of the Black population is a shared inequity across all homeless individuals, non-conforming gender identities serve as a significant intersectional factor, exposing Black transgender people and *travestis* to violence and social exclusion driven by both racial and gender-based discrimination^.^ 8

The majority of trans people and *travestis* reported being unemployed, although many engaged in some form of informal paid activity. Most had never held formal employment. Their labor context was predominantly characterized by self-employment, underemployment and informal work or complete exclusion from the labor market^.8,^ 20

The most common informal activities included prostitution and collecting and selling recyclable materials, often performed simultaneously. 

These activities reflect the challenges faced in securing formal employment, which frequently push transgender people and *travestis* into more accepting sectors such as compulsory sex work, beauty, entertainment, and the arts^.8,^ 20 The streets, whether as a venue for sex work or artistic performances, constituted a significant social space in their trajectories, including their political organization to advocate against violence^.9,21,^ 22

The majority of the transgender and *travesti* population experiencing homelessness reported having been sheltered in institutional housing facilities, which serve as temporary residences. Despite the high number of registrations in the Unified Registry, the majority of this population were not Bolsa Família beneficiaries, the main federal cash transfer program. According to the information on monthly income provided by trans people and *travestis* participating in the study, two of them were eligible for Bolsa Família Program (up to ¼ of the minimum wage)^.^ 23 The reasons for the absence of this benefit were not specified, but may include lack of documentation, social discrimination and lack of knowledge about their rights. Bureaucratic barriers, such as the mismatch of gender identity in official documents and insufficient training of service professionals, further contributed to the exclusion and marginalization of this group in accessing social assistance services^.8,^ 16

The homeless transgender and *travesti* population faced challenges in accessing health services, relying primarily on emergency care facilities (n=19), such as emergency rooms. This pattern reflects a tendency to seek care only in acute or life-threatening situations^.16,24,^ 25 A lack of understanding about primary healthcare as part of the emergency care network was evident, likely due to the breakdown in service provision at primary health centers and family health units, along with the misconception that primary care is limited to scheduled consultations^.16,^ 17

This disengagement was further exacerbated by institutional transphobia perpetuated by healthcare professionals, ranging from failure to acknowledge gender identity to disrespecting social names, undermining the quality of care for the specific health needs of transgender people and *travestis* ^.16,17,26-^ 28

The use of specialized mental health services was observed among trans people and *travestis*, particularly in psychosocial care center for alcohol and other drug use. This result corroborated the high rates of negative mental health outcomes, including substance use^.^ 29 It further emphasizes their detachment from health promotion and disease prevention initiatives offered by primary care services^.^ 30

Although homelessness itself exposes individuals to numerous rights violations, being transgender adds a layer of vulnerability, manifesting even before homelessness occurs and intensifying with this new condition. It is essential to adopt a political commitment to recognize the resilience of trans people and *travestis* in the face of marginalization, as expressed through their identity affirmation, organization, and social participation in rights advocacy.

Selection bias was a limiting factor in this study. The sample was obtained through convenience sampling, a common practice in similar studies due to the challenges in reaching the homeless population. Despite this limitation, the study stands out for its singular focus on the trans and *travesti* population in the context of homelessness, exploring a wide set of variables related to the sociodemographic profile and access to social assistance and health services. The sample of trans people and *travesti* (n=24) did not allow for statistical analyses of association. 

This study aims to contribute to the understanding of the trans and *travesti* population experiencing homelessness, addressing a gap that has been little explored in the international and national literature. The findings emphasize the need to improve specific public policies aimed at ensuring the basic rights of transgender people and *travestis*, such as healthcare, housing, and employment, to facilitate their transition out of homelessness. It is expected that these data will stimulate further debate on this topic and guide future research to provide more detailed information and robust data analyses on factors associated with morbidity and mortality in this population.

Combining quantitative and qualitative approaches is critical to understanding the personal, social, and contextual experiences contributing to the vulnerabilities faced by homeless transgender people and *travestis*. Ongoing education for health and social service professionals, grounded in scientific evidence and social movement guidelines, is essential. Reducing institutional violence, particularly barriers to access, and improving the quality of care for this population are key objectives.

In conclusion, the findings of this study reveal the profound vulnerability of homeless transgender people and *travestis*, who are subjected to multiple forms of discrimination and social exclusion. The intersection of social class, gender, and race exacerbates this situation, limiting access to essential services such as healthcare and employment. These results highlight the urgent need for specific public policies to safeguard basic rights, promote social inclusion, and combat transphobia. Future research should delve deeper into the lived experiences of these individuals to develop more effective interventions.
