# Supplementary material for: Transgender people and travestis experiencing homelessness in Salvador: a descriptive study on sociodemographic profile and access to social assistance and health services, Brazil, 2021 and 2022
Source: Epidemiol Serv Saude. 2025 Jan 13;33(spe1):e2024515. doi: 10.1590/S2237-96222024v33e2024515.especial.en (PMC11734597; doi:10.1590/S2237-96222024v33e2024515.especial.en)
Supplement: Translation

# Translation: Pessoas trans e travestis em situação de rua em Salvador: estudo descritivo sobre o perfil sociodemográfico e de acesso aos serviços socioassistenciais e de saúde, 2021 e 2022

**DOI:** 10.1590/S2237-96222024v33e2024515.especial.pt

**Authors:** Wiler de Paula Dias, Thayane Silva Nunes, Igor Myron Ribeiro Nascimento, Erik Asley Ferreira Abade, Lua Sá Dultra, Milena Lemos Marinho, Guilherme de Sousa Ribeiro, Joilda Silva Nery

**Affiliations:** 1 Universidade Federal da Bahia, Instituto de Saúde Coletiva, Salvador, BA, Brasil; 2 Universidade Federal da Bahia, Faculdade de Medicina, Salvador, BA, Brasil; 3 Fundação Oswaldo Cruz, Instituto Gonçalo Moniz, Salvador, BA, Brasil

**Keywords:** População em Situação de Rua, Pessoas Transgênero, Travestis, Vulnerabilidade Social, Inquérito Epidemiológico

## Abstract

**Objetivo:**

Descrever o perfil sociodemográfico e de acesso aos serviços socioassistenciais e de saúde entre pessoas trans e travestis em situação de rua em Salvador.

**Métodos:**

Trata-se de estudo transversal com 24 (4,5%) participantes que se autoidentificaram como pessoas trans ou travestis do total de 529 pessoas, com idade igual ou superior a 18 anos, presentes em logradouros públicos ou unidades de acolhimento institucional incluídas no inquérito. Os dados foram coletados entre 2021 e 2022, por meio de questionários estruturados.

**Resultados:**

Chegou-se à amostra majoritariamente composta por mulheres trans e travestis (n=18), jovens (n=13), negras (n=22), solteiras (n=18) e com ocupações informais (n=23), nas quais relataram sofrer violência (n=17). Foram encontradas dificuldades de acesso aos serviços de saúde e socioassistenciais frequente (n=15), devido à ausência de documentação, demora no atendimento e discriminação social/racial.

**Conclusão:**

Observa-se a vulnerabilização acentuada de pessoas trans e travestis em situação de rua, submetidas a múltiplas formas de discriminação e exclusão social.

## Contribuições do estudo

**Principais resultados:**

O perfil da amostra foi composto por mulheres trans, jovens, pretas, solteiras e com ocupação informal. A dificuldade de acesso aos serviços de saúde/socioassistenciais teve como principais causas a ausência de documentos e a discriminação social/racial.

**Implicações para os serviços:**

O estudo abordou o afastamento e as barreiras que pessoas trans e travestis enfrentam no acesso aos serviços de saúde e socioassistenciais. Ressaltou-se a importância do fortalecimento de estratégias que ampliem a rede de atenção e cuidado adequado.

**Perspectivas:**

Recomendam-se novos estudos que investiguem a possível maior susceptibilidade das pessoas trans e travestis à situação de rua e suas necessidades em saúde. A implementação de políticas públicas que atendam às demandas dessa população é urgente.

## INTRODUÇÃO 

A população em situação de rua é definida como grupo heterogêneo que compartilha a intersecção de fatores estruturais e pessoais, como exclusão econômica, racismo e rompimento de vínculos familiares, e a inexistência de moradia convencional regular, tendo a rua como espaço principal de reprodução social^.1,^ 2 A existência e o aumento da população em situação de rua não são ocorrências recentes. Isso é reflexo das desigualdades históricas e questões macroeconômicas que persistem como consequência desses fatores no sistema capitalista^.3-^ 5

No Brasil, em 2023, estimou-se que mais de 236 mil pessoas não tinham residência fixa e viviam nas ruas ou em unidades de acolhimento para a população em situação de rua. O perfil da população foi predominantemente composto por adultos com idades entre 30 e 49 anos (55%), do sexo masculino (87%), negros (68%) e com experiência pregressa de trabalho formal com carteira assinada (68%)^.^ 6

O rompimento com o padrão cisheteronormativo contribui significativamente para a desfiliação social e aumenta a possibilidade de ida para as ruas para a população LGBTQIAPN+, sobretudo para as pessoas trans e travestis^.7-^ 9 Conflitos familiares relacionados ao inconformismo ao sistema binário de sexo-gênero são apontados como a principal razão do afastamento. Isso reforça a discriminação por orientação sexual e identidade de gênero como determinante social de saúde^.10-^ 12

Pessoas não cisgênero, tanto no ambiente familiar quanto nas ruas, enfrentam vulnerabilidades que resultam em exclusões e negações ao longo de suas vidas. Tais pessoas são sujeitadas a contextos de desumanização e abjeção, bem como à maior propensão à violência física e sexual^.8,9,10,^ 13 Em 2023, no Brasil, foram registrados 136 casos de assassinato de travestis e mulheres trans, destacando-se mulheres negras, na faixa etária de 18 a 29 anos, muitas delas trabalhadoras do sexo, principalmente no Nordeste e no Sudeste do país^.^ 14 A falta de habitação segura foi agravante nessa exposição à violência^.13,^ 15

No campo da saúde, persistem desafios significativos que comprometem o acesso aos serviços e a qualidade da atenção à saúde ofertada. A insuficiente qualificação dos profissionais sobre as necessidades em saúde da população LGBTQIAPN+ e a permanência do preconceito social contra a população em situação de rua, além do estigma relacionado ao vírus da imunodeficiência humana (HIV), são algumas das razões que podem contribuir para o aumento da vulnerabilidade dessa população a agravos, doenças e desfechos desfavoráveis em saúde^.10,11,16,^ 17 São escassas as pesquisas relacionadas às condições de saúde e as limitações de acesso enfrentadas por pessoas trans e travestis, especialmente em contextos de maior vulnerabilidade como em situação de rua, visando promover o bem-estar físico e mental dessa população^.10,^ 17

Este estudo objetivou colaborar na construção de conhecimento sobre a temática. Buscou-se descrever o perfil sociodemográfico e de acesso a serviços socioassistenciais e de saúde entre pessoas trans e travestis em situação de rua em Salvador entre 2021 e 2022. 

## MÉTODOS

### Delineamento

Este trabalho originou-se do inquérito epidemiológico Condições de Saúde da População em Situação de Rua de Salvador – Bahia, 2021, estudo transversal que incluiu amostra de pessoas em situação de rua em Salvador, Bahia. 

### Contexto

A coleta de dados do inquérito ocorreu entre setembro de 2021 e fevereiro de 2022. Os locais onde foi realizada a inclusão dos participantes foram definidos após a identificação das unidades de acolhimento institucionais vinculadas à Secretaria de Promoção Social, Combate à Pobreza, Esporte e Lazer da Prefeitura Municipal de Salvador (17 unidades de acolhimento institucionais) e o mapeamento dos logradouros públicos (40 logradouros) onde se concentram pessoas em situação de rua e onde atuam as cinco equipes de consultório na rua do município: Centro Histórico-Gamboa, Centro Histórico-Pelourinho, Itapuã, Itapagipe e Brotas (Figura 1). 

**Figura 1:** Cobertura dos distritos sanitários, pontos de concentração da população em situação de rua e unidades de acolhimento institucional, Salvador, 2021-2022

As unidades de acolhimento institucionais de Salvador foram divididas por tipologias (femininas, masculinas, mistas, casal, idosos e família). Com o intuito de garantir a diversidade da população em situação de rua entrevistada, sobretudo por tratar-se de grupo heterogêneo, garantiu-se que, pelo menos, uma unidade de acolhimento institucional de cada tipo fosse incluída no estudo, sendo realizado sorteio por subtipo. Foram definidas de duas a três unidades de acolhimento institucionais para cada consultório na rua, em ordem pré-determinada. 

Na rua, os pontos de concentração da população em situação de rua foram sorteados aleatoriamente para cada consultório na rua, sendo definida ordem de prioridade para a realização do inquérito. Todos os pontos foram visitados em todas as áreas dos consultórios na rua. Como o número mínimo de participantes não foi atingido com apenas uma visita por local, houve visitas repetidas a esses territórios, seguindo ainda o sorteio de priorização. Por tratar-se de população de difícil acesso, a inclusão foi oportunizada a todos interessados presentes nos locais, desde que atendessem aos critérios de elegibilidade previamente estabelecidos.

### Participantes 

A população de referência alvo para inclusão no inquérito consistiu em pessoas identificadas como em situação de rua. As características destes foram correspondentes às descritas na Política Nacional para a População em Situação de Rua: grupo populacional heterogêneo, em pobreza extrema, com os vínculos familiares interrompidos ou fragilizados, cuja moradia inexistia e “que utiliza os logradouros públicos e as áreas degradadas como espaço de moradia e de sustento, de forma temporária ou permanente, bem como as unidades de acolhimento para pernoite temporário ou como moradia provisória”^.^ 1

Os critérios de elegibilidade para participar do estudo foram: autodeclarar-se pessoa em situação de rua, a partir da definição já referida, não apresentar deficiência intelectual, permanente ou transitória, associada ou não ao uso de substâncias psicoativas, que a impedisse de responder ao questionário, e não apresentar sintomas respiratórios há menos de 10 dias, pelo risco de transmissão de covid-19 à equipe de pesquisa. Os sintomáticos respiratórios foram encaminhados para atendimento, conforme o protocolo municipal vigente à época.

### Variáveis

Para análises, foram utilizadas variáveis agrupadas em três grandes blocos, conforme a seguir.

Sociodemográficas e econômicas: gênero (mulher trans, travesti, homem trans, pessoa não binária, homem cis, mulher cis), faixa etária (18-29 anos, 30-39 anos, 40-49 anos, ≥50 anos), raça/cor da pele (preta, branca, parda, amarela, indígena), orientação sexual (heterossexual, *gay*, lésbica, bissexual, assexual, pansexual), escolaridade (ensino superior completo/incompleto, ensino médio completo/incompleto, ensino fundamental completo/incompleto, sem escolaridade), sabe ler (não; sim, sem dificuldades; sim, com dificuldades), sabe escrever (não; sim, sem dificuldades; sim, com dificuldades), estado civil (solteira/o, casada/o legalmente, união estável, separada/o ou divorciada/o, viúva/o), naturalidade (Salvador e Região Metropolitana de Salvador, outras localidades), tempo em situação de rua (<1 ano, 1-4 anos, 5-9 anos, ≥10 anos), renda mensal (<¼ de salário mínimo, ¼-½ de salário mínimo, ½-¾ de salário mínimo, >3/4 de salário mínimo), possui alguma deficiência (não, sim) e distrito sanitário (Centro Histórico, Itapuã, Itapagipe, Brotas).
Ocupacionais: trabalha atualmente (não, sim), realiza algum tipo de trabalho informal (não, sim), trabalhos informais simultâneos (1, 2, ≥3), carteira de trabalho assinada (sim, atualmente; sim, no passado; nunca), já foi impedido de trabalhar (não, sim) e já sofreu violência durante o trabalho (não, sim).
Acesso aos serviços socioassistenciais e de saúde: histórico de acolhimento institucional (não, sim), registro no Cadastro Único (não, sim), recebe Bolsa Família (não, sim), dificuldade de acesso aos postos de saúde (não, sim), onde costuma buscar atendimento (variável politômica), barreiras de acesso aos serviços de saúde (variável politômica), serviços de assistência social frequentados no último ano (variável politômica), acompanhamento em serviços de saúde mental (variável politômica) e atendimentos de saúde na rua (variável politômica).

### Fontes de dados e mensuração

A coleta de dados foi realizada por equipe composta por profissionais dos consultórios na rua, trabalhadores dos equipamentos de saúde mental, direitos humanos e assistência social, estudantes universitários e voluntários. Após o consentimento dos participantes, os questionários foram aplicados utilizando aparelhos eletrônicos (celulares e *tablets*), por meio do *software* Research Electronic Data Capture (RedCap).

### Tamanho do estudo

A amostra final deste estudo foi constituída por 24 participantes (4,5%) que se autoidentificaram como pessoas trans ou travestis, de um total de 529 incluídas no inquérito sobre pessoas em situação de rua. Para inclusão na amostra, foi realizado o questionamento “Como você se reconhece em relação ao seu gênero? (Qual a sua identidade de gênero?)”. Foram consideradas válidas as respostas: mulher trans, travesti, homem trans, pessoas não binárias e outros.

A amostra mínima do inquérito sobre pessoas em situação de rua foi calculada em 560 participantes, com base na estimativa de prevalência de 50% para múltiplos desfechos de saúde, nível de confiança de 95% e erro amostral de 4%, sem ajustes para efeito do desenho.

### Análise de dados

Realizou-se análise descritiva do perfil dos participantes por meio de frequências absolutas, por categorias. A organização e a limpeza do banco de dados, bem como a condução das análises estatísticas, foram realizadas utilizando o *software R*, versão 4.2.3 (x64). A construção das ilustrações foi elaborada pelos autores, fundamentando-se nos dados do inquérito Condições de Saúde da População em Situação de Rua de Salvador – Bahia, 2021. 

### Aspectos éticos

A pesquisa foi conduzida conforme as recomendações da Resolução nº 466/2012, do Conselho Nacional de Saúde, aprovada pelo Comitê de Ética em Pesquisa do Instituto Gonçalo Moniz/Fiocruz (CAAE: 42517021.0.0000.0040), e do Parecer nº 5.138.407, aprovado em 1º de dezembro de 2021. Cada participante foi informado sobre objetivos, procedimentos e riscos. Ao aceitarem a participação no inquérito, assinaram e receberam uma cópia do termo de consentimento livre e esclarecido da pesquisa.

## RESULTADOS

Entre as 529 pessoas em situação de rua entrevistadas, 24 identificaram-se como pessoas trans e travestis, correspondendo a 4,5% do total da amostra. A população trans e travesti em situação de rua entrevistada foi composta por maioria de mulheres trans (n=15), jovens entre 18 e 29 anos (n=13), heterossexuais (n=12), autodeclaradas pretas e pardas (n=22), solteiras (n=18), com renda média mensal até ½ salário-mínimo (n=9), naturais de Salvador ou região metropolitana (n=20) e sem deficiência física ou mental (n=21). A maioria possuía até o nível fundamental completo ou incompleto (n=14). Muitas estavam em situação de rua há menos de um ano (n=14). O distrito sanitário Centro Histórico concentrou o maior número de entrevistados (n=11) (Tabela 1).

**Tabela 1:** Descrição de pessoas trans e travestis em situação de rua segundo características socioeconômicas e demográficas, Salvador, 2021-2022 (n=24)

**Variáveis**	**n**
Gênero autorreferido	
Mulher trans	15
Travesti	3
Homem trans	4
Pessoa não binária	2
**Faixa etária (anos)**	
18-29	13
30-39	6
40-49	4
≥50	1
**Raça/cor de pele**	
Preta	14
Branca	2
Parda	8
**Orientação** **sexual autorreferida**	
Heterossexual	12
Gay	6
Lésbica	2
Bissexual	2
Assexual	1
Pansexual	1
**Escolaridade**	
Sem escolaridade	-
Fundamental completo ou incompleto	14
Ensino médio completo ou incompleto	8
Superior completo ou incompleto	2
**Sabe ler**	
Não	3
Sim, com dificuldades	5
Sim, sem dificuldades	16
**Sabe escrever**	
Não	1
Sim, com dificuldades	9
Sim, sem dificuldades	14
**Estado** **civil**	
Solteiro(a)	18
Casado(a) legalmente	-
União estável	4
Separado(a) ou divorciado(a)	1
Viúvo(a)	1
**Naturalidade**	
Salvador e região metropolitana de Salvador	20
Outra localidade	4
**Tempo em** **situação de rua (anos)**	
<1	14
1-4	3
5-9	3
≥10	4
**Renda mensal** ^a^ **(salário mínimo)**	
<¼	2
¼-½	7
½-¾	1
>¾	4
**Possui alguma deficiência** ^b^	
Não	21
Sim	2
**Distrito sanitário**	
Centro Histórico	11
Itapuã	5
Itapagipe	4
Brotas	4

a) Com base no salário-mínimo de 2021; b) Valor absoluto de respostas alterou-se em função da perda de informações (respostas em branco) na variável analisada (n=23).

Mais da metade (n=14) da população trans e travestis em situação de rua afirmou já ter sido abrigada em unidades de acolhimento institucionais. Quinze pessoas informaram ter registro no Cadastro Único para Programas Sociais. A maior parte não recebia Bolsa Família (n=15). Considerando os últimos 12 meses prévios à realização do inquérito, os serviços mais frequentados foram o Centro de Referência Especializado para População em Situação de Rua (n=13), seguido pelo Centro de Referência da Assistência Social (n=10), pelo Centro de Referência Especializado de Assistência Social (n=2) e pelo Centro de Convivência (n=2). 

Dezoito pessoas declararam não estar trabalhando, mas a alta participação em atividades informais (n=23) foi notada. Observou-se a realização de uma única atividade por vez (n=18) quanto à frequência de realização de trabalho informal. A maioria nunca teve a carteira assinada (n=20). Grande parte (n=17) das pessoas trans e travestis relataram ter sofrido algum tipo de agressão, ameaça ou violência durante a execução de suas atividades laborais (Tabela 2). 

**Tabela 2:** Descrição das pessoas trans e travestis em situação de rua, segundo variáveis ocupacionais e de acesso aos serviços socioassistenciais, Salvador, 2021-2022 (n=24)

**Variáveis**	**n**
Trabalha atualmente	
Não	18
Sim	6
**Realiza algum tipo** **de trabalho informal**	
Não	1
Sim	23
**Número de trabalhos informais** **simultâneos** ^a^	
1	18
2	4
≥3	1
**Trabalha ou j** **á trabalhou** **com registro em carteira de trabalho**	
Não (nunca)	20
Sim (no passado)	4
Sim (no presente)	-
**Já foi impedido** **de trabalhar**	
Não	15
Sim	9
**Já sofreu violência durante o** **trabalho**	
Não	7
Sim	17
**Tipos de trabalhos informais realizados** ^b^	
Catador(a)	5
Profissional do sexo	10
Guardador(a)/limpador(a) de carro	3
Vendedor(a)	8
Serviços gerais	1
Manicure e pedicure	2
**Serviços de assistência social frequentados no último ano** ^b^	
CRAS	10
CREAS	2
Centro POP	13
Centro de convivência e fortalecimento de vínculos	2
Nenhum	2
**Histórico de acolhimento institucional**	
Não	10
Sim	14
**Registro no Cadastro** **Único**	
Não	9
Sim	15
**Recebe** **Bolsa Família**	
Não	15
Sim	9

a) Valor absoluto de respostas alterou-se em função da perda de informações (respostas em branco) na variável analisada (n=23); b) O N total reflete a possibilidade de múltiplas opções de resposta; c) CRAS – centro de referência da assistência social; CREAS – centro de referência especializado da assistência social; e Centro POP – centro de referência especializado para população em situação de rua.

 Os resultados relacionados ao acesso aos serviços de saúde por pessoas trans e travestis em situação de rua indicam que a unidade de pronto atendimento foi o principal local de busca por atendimento (n=19), seguido pelo consultório na rua (n=9) e pelas unidades básicas de saúde e saúde da família (n=8), sendo hospitais os espaços menos frequentados (n=4). Não houve relatos de busca por atendimento em policlínicas ou unidades especializadas. A maioria relatou alguma dificuldade de acesso a unidades de saúde (n=12). Identificaram-se como motivos da dificuldade de acesso a ausência de documento de identificação (n=3), a falta do cartão nacional de saúde (n=5) e a demora no atendimento (n=6). As discriminações sociais (n=4) e raciais (n=1) também foram apontadas pelas respondentes (Tabela 3). 

**Tabela 3:** Descrição das pessoas trans e travestis em situação de rua segundo acesso aos serviços de saúde, Salvador, 2021-2022 (n=24)

Variáveis	n
**Já enfrentou dificuldade de acesso a unidades de saúde ^a^**	
Não	11
Sim	12
**Onde costuma buscar atendimento de saúde** ^b^	
UBS/USF	8
CnaR	9
Unidade de pronto atendimento	19
Policlínicas ou centro de especialidades	-
Hospitais	4
Não costuma ir a serviços de saúde	-
Outros^c^	1
**Barreiras de acesso aos serviços de saúde** ^b^	
Ausência de documento de identificação	3
Ausência de cartão do Sistema Único de Saúde	5
Não ter agendado consulta previamente	1
Discriminação racial	1
Discriminação social	4
Demora no atendimento	6
**Já fez** **acompanhamento em serviços de saúde mental** ^b^	
Nunca	7
CAPSad ou CAPSad III	7
CAPSia/CAPS II/CAPS III	1
Comunidade terapêutica ou centro de recuperação	5
Internação em hospitais ou Cata	1
Ambulatório ou Cetad	2
Outros^d^	1
**Tipo de** **atendimentos de saúde na rua recebido** ^b^	
Consulta médica ou de enfermagem	12
Vacinação	7
Aplicação de medicamentos	3
Curativo	4
Teste rápido de ISTs	7
Coleta de escarro para investigação de tuberculose	2
Atendimento por profissionais de saúde (exceto médicas(os) ou enfermeiras(os))	9

a) Variável dicotômica. Valor absoluto de respostas alterou-se em função da perda de informações (respostas em branco) na variável analisada (n=23); b) O N total reflete a possibilidade de múltiplas opções de resposta; c) Faculdade de medicina; d) Redutores de danos, psicólogos, terapeutas ocupacionais, assistentes sociais e educadores físicos; e) UBS/USF – unidade básica de saúde ou unidade de saúde da família; CnaR – consultório na rua; CAPSad – centro de atenção psicossocial álcool e drogas; CAPsia – centro de atenção psicossocial infância e adolescência; Cata – centro de acolhimento e tratamento de alcoolistas; Cetad – centro de estudos e terapia do abuso de drogas; e ISTs – infecções sexualmente transmissíveis.

Muitos dos participantes nunca realizaram acompanhamento em serviços de saúde mental (n=7). A mesma quantidade (n=7) indicou ter feito acompanhamento no centro de atenção psicossocial álcool e outras drogas. Comunidades terapêuticas ou centros de recuperação relacionados ao abuso de álcool e outras drogas (n=5) também foram utilizados.

As consultas médicas ou de enfermagem foram as mais frequentes (n=12) nos atendimentos de saúde realizados na rua. Destacou-se também o atendimento com outras categorias de profissionais de saúde. A vacinação (n=7), os testes rápidos de infecções sexualmente transmissíveis (n=7) e a aplicação de medicamentos (n=3) foram os procedimentos realizados mais frequentes.

## DISCUSSÃO

Os resultados desta pesquisa indicaram a maior vulnerabilidade de mulheres trans e travestis a violências e desfechos sociais desfavoráveis, mesmo quando comparadas aos demais subgrupos da população LGBTQIAPN+^.7,8,9,11,^ 14

Estimou-se que 1,9% da população brasileira adulta se reconheceu como pessoa trans ou travestis^.^ 18 A proporção de 4,5% encontrada neste estudo reforçou o efeito da exclusão social enfrentada por pessoas trans e travestis em situação de rua, fruto do processo de marginalização social vivenciado por essa população pelo rompimento com a norma cisheterossexual^.7-9,11,12,^ 17

Desde a gestação até o momento em que expressam sua identidade a qual questiona o sistema sexo-gênero e as conseguintes expectativas de comportamentos desejáveis, a invisibilidade sobre a existência e a luta das pessoas trans e travestis torna-se um mecanismo, associando-as a “o outro”, “o estranho”, “o abjeto”, que passa a existir apenas para que seja eliminado^.^ 13 “É um processo de dar vida, através do discurso, para imediatamente matá-lo”^.^ 19

A maior parte das pessoas trans e travestis se autodeclarou negra, com predominância de pessoas pretas. Esse dado refletiu a própria formação histórica da população em situação de rua, remetendo aos ex-escravizados. Estes, após a abolição da escravatura, não tiveram apoio jurídico-legal ou reparação e encontraram nos espaços públicos, sobretudo urbanos, sua residência^.4,^ 5 Embora pertencer à população negra tenha sido fator de iniquidade comum à toda população em situação de rua, a identidade de gênero não conforme configurou-se como elemento de intersecção relevante, expondo pessoas trans e travestis negras a situações de violência e exclusão social motivadas por fatores raciais, mas também pelo gênero^.^ 8

A maioria das pessoas trans e travestis afirmou não trabalhar, embora tenha relatado estar envolvida em alguma forma de atividade informal remunerada. Grande parte nunca teve emprego formal. Observou-se o contexto laboral predominantemente caracterizado pelo autoemprego, subemprego e trabalho informal ou a exclusão total do mercado de trabalho^.8,^ 20

As atividades informais mais comuns foram a prostituição e a venda e catação de materiais recicláveis, por vezes desempenhadas de forma simultânea. Isso refletiu as dificuldades enfrentadas na busca por empregos formais, que acabam direcionando as pessoas trans e travestis a setores mais receptivos, como prostituição compulsória, beleza, entretenimento e arte^.8,^ 20 O cenário da rua, tanto na dimensão da prostituição como na dimensão das apresentações artísticas, foi um espaço social presente na trajetória desse grupo, inclusive para sua organização política na luta pelo fim da violência^.9,21,^ 22

A maioria da população trans e travestis em situação de rua afirmou já ter sido abrigada em unidades de acolhimento institucionais, locais que atuam como moradia provisória. Apesar da alta presença de registro no Cadastro Único, a maior parte dessa população não recebia Bolsa Família, principal programa federal de transferência de renda. De acordo com as informações sobre renda mensal fornecidas pelas pessoas trans e travestis participantes, duas delas estavam aptas para receber o Bolsa Família (até ¼ de salário mínimo)^.^ 23 As razões para ausência do benefício não foram especificadas, mas falta de documentação, discriminação social e falta de conhecimento sobre os direitos são situações que podem ser incluídas. Barreiras burocráticas, como a falta de adequação da identidade de gênero nos documentos e a de capacitação dos profissionais, contribuíram para a exclusão e marginalização desse grupo no acesso a serviços socioassistenciais^.8,^ 16

A população trans e travesti em situação de rua enfrentou dificuldades de acesso a serviços de saúde, recorrendo majoritariamente a unidades de urgência e emergência (n=19), como as unidades de pronto atendimento. Esse padrão refletiu a busca por cuidados apenas em situações agudas ou de risco iminente de morte^.16,24,^ 25 Evidenciou-se o possível desconhecimento da atenção primária à saúde como parte da rede de atenção à urgência e emergência, resultado da desestruturação no acolhimento nas unidades básicas de saúde e saúde da família e do equívoco de que a atenção primária se limitava a consultas agendadas^.16,^ 17

Esse afastamento também foi intensificado pela transfobia institucional, reproduzida por profissionais de saúde. Isso aconteceu desde a falta de reconhecimento da identidade de gênero até o desrespeito ao nome social, comprometendo a qualidade do atendimento às necessidades específicas de saúde de pessoas trans e travestis^.16,17,26-^ 28

A utilização de serviços especializados em saúde mental por pessoas trans e travestis foi observada, com destaque para o centro de atenção psicossocial álcool e outras drogas. Este resultado corroborou as altas taxas de desfechos negativos em saúde mental, incluindo o uso de substâncias^.^ 29 Reforçou-se o afastamento das ações de promoção da saúde e prevenção de doenças ofertadas pela atenção primária^.^ 30

Ainda que o estar em situação de rua seja condição suficiente para vivenciar situações inúmeras de violações de direitos, ser uma pessoa trans em situação de rua lhe acrescenta uma camada de vulnerabilidade, que é deflagrada desde antes da ida para as ruas, sendo agravada com essa nova condição. É essencial assumir o compromisso político em reconhecer a resiliência das pessoas trans e travestis diante de suas experiências marginalizadas, expressa pela afirmação de suas identidades, organização e participação social nas lutas por direitos. 

O viés de seleção foi fator limitante deste estudo. A amostra foi selecionada por conveniência, prática comum em estudos similares, em razão da dificuldade em alcançar o segmento populacional em situação de rua. Apesar desta limitação, o estudo se destaca por seu foco singular na população trans e travestis no contexto da situação de rua, explorando um amplo conjunto de variáveis relacionadas ao perfil sociodemográfico e de acesso aos serviços socioassistenciais e de saúde. A amostra de pessoas trans e travestis (n=24) não permitiu a realização de análises estatísticas de associação. A descrição do perfil sociodemográfico e de acesso a serviços socioassistenciais e de saúde auxiliaram na compreensão das necessidades em saúde e demonstraram a relevância de novos estudos, em nível nacional ou regional, com essa população. 

Este estudo visa contribuir para o conhecimento sobre a população trans e travesti em situação de rua, preenchendo a lacuna pouco explorada na literatura internacional e nacional. Os resultados indicam a necessidade de aprimorar políticas públicas específicas de cidadania para população trans e travesti, a fim de antecipar a saída das ruas, a partir da garantia de direitos básicos, como saúde, moradia e emprego. Espera-se que os dados apresentados possam estimular o debate sobre o tema e sugerir direções para pesquisas futuras, que forneçam informações mais detalhadas e análises de dados mais robustas sobre fatores associados à morbimortalidade nessa população. 

Cabe ressaltar a importância de combinar abordagens quantitativas e qualitativas visando compreender as experiências pessoais, sociais e contextuais que contribuem para as vulnerabilidades enfrentadas pela população trans e travesti em situação de rua. Destaca-se a importância da educação permanente para profissionais de saúde e assistência social, com base na produção científica e nas diretrizes dos movimentos sociais. A redução das violências institucionais, sobretudo as barreiras de acesso, e a melhoria do cuidado prestado a essa população são as finalidades visadas.

Em conclusão, os resultados deste estudo evidenciam a profunda vulnerabilidade de pessoas trans e travestis em situação de rua, submetidas a múltiplas formas de discriminação e exclusão social. A intersecção entre classe social, gênero e raça agrava essa situação, limitando o acesso a serviços essenciais como saúde e trabalho. Os achados destacam a urgência de políticas públicas específicas para essa população, que garantam direitos básicos, promovam a inclusão social e combatam a transfobia. Futuras pesquisas devem aprofundar a compreensão das experiências vividas por essas pessoas, com o objetivo de desenvolver intervenções mais eficazes.
